# Gonadotrophin releasing hormone antagonist in IVF/ICSI

**DOI:** 10.4103/0974-1208.39594

**Published:** 2008

**Authors:** Kamath MS, Mangalraj AM, Muthukumar KM, George K

**Affiliations:** Reproductive Medicine Unit, Christian Medical College Hospital, Vellore - 632 004, Tamil Nadu, India

**Keywords:** Assisted reproduction, Gonadotrophin releasing hormone antagonists, ICSI, IVF

## Abstract

**OBJECTIVE:**

To study the efficacy of gonadotrophin releasing hormone (GnRH) antagonist in In-vitro-fertilization/Intracytoplasmic sperm injection (IVF/ICSI) cycles.

**TYPE OF STUDY:**

Observational study.

**SETTING:**

Reproductive Medicine Unit, Christian Medical College Hospital, Vellore, Tamil Nadu.

**MATERIALS AND METHODS:**

GnRH antagonists were introduced into our practice in November 2005. Fifty-two women undergoing the antagonist protocol were studied and information gathered regarding patient profile, treatment parameters (total gonadotrophin dosage, duration of treatment, and oocyte yield), and outcomes in terms of embryological parameters (cleavage rates, implantation rates) and clinical pregnancy. These parameters were compared with 121 women undergoing the standard long protocol. The costs between the two groups were also compared.

**MAIN OUTCOME:**

Clinical pregnancy rate.

**RESULTS:**

The clinical pregnancy rate per embryo transfer in the antagonist group was 31.7% which was comparable to the clinical pregnancy rate in women undergoing the standard long protocol (30.63%). The costs between the two groups were comparable.

**CONCLUSIONS:**

GnRH antagonist protocol was found to be effective and comparable to the standard long protocol regimen. In addition it was simple, convenient, and patient friendly.

Gonadotrophin releasing hormone agonists (GnRH-a) were introduced into IVF protocols in the 1980s to downregulate the pituitary gland so as to prevent a premature luteinizing hormone (LH) surge. This resulted in decreased cycle cancellation and increased clinical pregnancy rates.[1,2] It also allowed programming of the IVF cycle for both physician and patient convenience. Several downregulation protocols are currently in use, but the long protocol remains the most effective and frequently used regimen.[3]

The initial flare effect of the agonist necessitates longer duration of treatment to achieve adequate downregulation. Other drawbacks of this regimen include greater gonadotrophin requirement and profound hypoestrogenic symptoms seen in some patients.[4]

GnRH antagonists competitively block pituitary receptor sites and induce a rapid and reversible suppression of gonadotrophins.[5] Reductions in both duration of therapy and gonadotrophin requirement make its use attractive. Other potential benefits include lower risk of severe ovarian hyperstimulation syndrome (OHSS), absence of estrogen deprivation symptoms, and a more patient friendly protocol.[6]

In assisted reproduction, the only outcome parameters of interest to both physician and client are the live birth and the clinical pregnancy rates. In a Cochrane review comparing the GnRH antagonist to the agonist long protocol, there were significantly fewer pregnancies in the antagonist group. However, with the use of antagonists there was a significant reduction in incidence of ovarian hyperstimulation, duration of cycle, and gonadotrophin requirement.[4] It is unclear whether the decline in pregnancy rates is due to an initial learning curve problem or if it is due to an intrinsic effect on folliculogenesis or endometrial receptivity.[7,8]

The potential advantages of the antagonist regimen prompted us to evaluate its role in our IVF program.

## MATERIALS AND METHODS

Women undergoing IVF/ICSI cycle at the Reproductive Medicine Unit, Christian Medical College Hospital, Vellore between November 2005 to October 2006 were studied. Initial selection was confined to women in the older age group and prior poor responders. Due to the convenience of the antagonist protocol, recruitment was later extended to included women who did not live close to the IVF unit.

Fifty-two women were invited to participate in the study, each undergoing one cycle of IVF/ICSI.

Cycle regulation was carried out in the cycle prior to the scheduled IVF with the oral contraceptive pill. Purified follicle stimulation hormone was started from the first or second day of flow in a dose appropriate to age and body weight. Women were scanned after the fourth dose onwards and the antagonist was added to the protocol once the lead follicle reached a size of 14 mm or more (multiple dose, flexible regime). The daily dose schedule of 0.25 mg of antagonist was given till the day of human chorionic gonadotrophin (hCG) trigger.

Women were scheduled for oocyte retrieval once at least three follicles reached a size of 18 mm or more. Transvaginal oocyte retrieval was planned 35 h after Inj hCG 5000 I.U was given.

Oocyte retrieval was done under conscious sedation using IV pethidine, midazolam, and fentanyl in titrated doses.

The retrieved oocytes were incubated for 3-4 h in fertilization medium and then depending upon situation (indication, number of oocytes, and previous fertilization rates) decision for IVF or ICSI was taken. Group culture and short incubation (2 h) were followed for IVF.

Denudation of the oocytes was carried out (both mechanical and enzymatic) before ICSI was done. The oocytes were incubated overnight in mini incubator with triple gas mixture and observed after 16-18 h postinsemination/injection for fertilization (presence of two pronucleus (PN) and two Polar bodies). The fertilized oocytes were transferred into cleavage medium, incubated, and observed for cleavage on day 2. The embryos were reviewed on day 3 and if four or more grade 1 embryos were obtained, they were transferred into blastocyst medium and cultured to day 5 for blastocysts.

The embryo transfers were done using Sydney IVF (SIVF) catheters on day 2, 3, or day 5 depending upon number and grade of embryos. Not more than three embryos were transferred in any one cycle.

Luteal support was given in the form of micronized vaginal progesterone pessaries in a dose of 400 mg twice daily for 18 days postoocyte retrieval. In addition 100 mg intramuscular (IM) progesterone was administered twice weekly.

Serum beta hCG was done on 18^th^ day following oocyte retrieval and if positive, a transvaginal ultrasound was done 10 days later to detect and confirm intrauterine pregnancy.

The primary outcome was clinical pregnancy rate per embryo transfer (Defined as presence of gestational sac with a fetal pole with cardiac activity on transvaginal ultrasound.).

The secondary outcomes included; total gonadotrophin usage, mean duration of stimulation, mean duration of antagonist and number of mature oocytes retrieved.

Fertilization rate: Cleavage rate, number of grade 1 embryos on day 3, implantation rate, cost per cycle.

Fertilization rate was defined as total number of fertilized oocytes by total number of mature oocytes retrieved.

Cleavage rate was defined as total number of day 2 embryos by total number of fertilized oocytes.

Embryos with at least eight distinct blastomeres and with <10% fragmentation were defined as grade 1.

Implantation rate was defined as number of gestational sacs determined by ultrasound by number of embryos transferred.

Patient profiles together with the primary and secondary outcomes were compared to the standard long protocol regimen being followed in our center during the same period of time. A total of 121 women were enrolled in the long protocol group. Except for the use GnRH agonist and recombinant follicle stimulating hormone (FSH), principles of clinical and laboratory management were similar.

The data were collected and the results were analyzed using SPSS software.

Student *t*-test was used to compare the baseline characteristics between two groups and the main outcomes were compared using the χ^2^-test.

## RESULTS

In antagonist group of the 52 cycles, 7 cycles were cancelled due to poor response, in three cases no oocytes were retrieved and in one fertilization failure occurred. A total of 41 embryo transfers were done and 13 clinical pregnancies were achieved (31.70%).

In the agonist group of the 121 cycles, 2 cycles were cancelled due to poor response, in five cases no oocytes were retrieved and in three fertilization failure occurred. A total of 111 embryo transfers were done and 34 clinical pregnancies were achieved (30.63%).

Patient profile, primary, and secondary outcomes were compared with the agonist long protocol as shown in Table 1.

**Table 1:** Comparison of antagonist and agonist cycles (patients profile, laboratory parameters, and clinical outcome)

Variable	Antagonist N = 52	Agonist N = 121	*P* value
	Mean (SD)	Mean (SD)	
Age (years)	33.75 (5.255)	30.93 (3.833)	0.00
Body mass index (BMI) (kg/m^2^)	25.75 (4.305)	25.09 (3.270)	0.312 (NS)
Serum FSH	6.388 (2.521)	6.020 (2.726)	0.495 (NS)
hMG/R-FSH	3456.54 (1629)	2287 (981.81)	0.00
Total number of days	10.21 (3.031)	9.96 (1.989)	0.517 NS)
Total number of M2	6.41 (5.888)	8.45 (6.559)	0.036 (NS)
Fertilization rates^a^	65.9% (182/276)	56.6% (570/1006)	0.006
Cleavage rates^a^	94.5% (172/182)	94.2% (h537/570)	0.803 (NS)
Day 3 grade 1	4.40 ± (2.898)	3.607 (2.469)	0.264 (NS)
Implantation rates^a^	21.5% (22/102)	22.9% (35/153)	0.774 (NS)
Pregnancy rates^a^	31.70% (31/41)	30.6% (34/111)	0.597 (NS)

a Value in percentages

Cost per cycle and comparison with the GnRH agonist protocol are shown in Table 2.

**Table 2:** Cost analysis for antagonist and agonist cycles

Variables	Antagonist group (Rs)	Agonist group (Rs)
Gonadotrophin used	34,560	36,592
Downregulation	8000	1000
14 days extra stay for downregulation @ Rs 500/day	NA	7000
Total	44,560	46,592

## DISCUSSION

Downregulation with GnRH agonists is a well-established practice in IVF. The flare effect due to its mechanism of action, long duration of treatment, increased requirement of gonadotrophins, and side effects due to the induced hypoestrogenic state are some of its main draw backs. With the introduction of the GnRH antagonist, it was hoped that many of these problems could be avoided and the IVF treatment protocol could be simplified.

Theoretically, GnRH antagonists are attractive in poor responders because their initiation occurs after the commencement of gonadotrophin stimulation, thus presumably minimizing their impact on early follicular recruitment. Some prospective randomized trials comparing antagonists with other protocols in poor responders have shown similar IVF outcomes in both groups.[9,10] However, the advantage of antagonists may lie in the ability to assess ovarian reserves immediately prior to deciding whether or not to initiate gonadotrophin stimulation. The ability to respond to cycle-to-cycle variation in antral follicle counts may allow the optimization of oocyte yield and reduce cycle cancellation rates.[11–14]

Many studies have been carried out comparing efficiency of antagonist regimens as compared to the standard long protocol. The Cochrane review comparing the long protocol with antagonist found significantly fewer pregnancies in antagonist group (odds ratio [OR] 0.78, 95% confidence intervel [CI] 0.62, 0.97). There was also significant reduction in incidence of OHSS (relative risk 0.36, 95% CI 0.16, 0.80) using antagonist regimen.[4] In women with polycystic ovarian syndrome (PCOS) this would be a safer drug to use. Moreover, the antagonist protocol allows the use of a single dose of GnRH agonist to trigger maturation avoiding the use of hCG thus further reducing the OHSS risk.[15]

The reason for the decline in pregnancy rates is unclear, but could be due to an initial learning curve problem with the introduction of GnRH antagonist. An intrinsic effect on folliculogenesis or endometrial receptivity is also speculated.[16,17]

In India assisted reproduction is self-financed and hence cost is a major factor to be considered, when introducing new drugs. This concern prompted us to use the more inexpensive option of purified gonadotrophins. Moreover, urinary products have been found to be as efficacious as the recombinant product.[18]

We evaluated 52 women who underwent IVF/ICSI cycle using the antagonist protocol. Although we initially recruited women expected to be poor responders, the indications were subsequently liberalized. This resulted in the study group having a mean age significantly higher than the comparison group. The higher gonadotrophin use in the antagonist group could be due to either of the differences mentioned above (mean age/differences in gonadotrophin used). Although the fertilization rate was significantly better in the antagonist group, the cleavage rates, number of grade 1 embryos on day 3, and implantation rate were comparable. The clinical pregnancy rates between the two groups were not significantly different (31.70% vs. 30.63%).

The mean cost of medication per cycle is almost similar. However, the use of the agonist necessitates the patient to stay an extra 14 days in close proximity to the IVF unit. This is especially important for centers with an influx of women residing at places away from the unit. The extra cost of the antagonist is offset by the reduction in the duration of stay.

The main drawbacks of this study are the possibility of selection bias and the difference in the type of gonadotrophins used. However, this was a pilot venture to introduce the antagonist into our practice.

In conclusion, the antagonist protocol was found to be convenient and suitable for women residing at a distance from the IVF unit. We were able to demonstrate comparable pregnancy rates when antagonist protocol was compared to the standard agonist protocol. The costs are similar.
